# Long-Term Outcome After Intensive Stroke Rehabilitation

**DOI:** 10.3390/jcm15093375

**Published:** 2026-04-28

**Authors:** Marilena Geißler, Anika Müller, Thomas Brauner, Aline Weidlich, Imanuel Dzialowski

**Affiliations:** 1 Medizinische Fakultät, TU Dresden, 01307 Dresden, Germany; marilena.geissler@mailbox.tu-dresden.de; 2 Elbland Reha Großenhain, 01558 Großenhain, Germany; anika.mueller@elbland-reha.de (A.M.);

**Keywords:** ischemic stroke, neurological rehabilitation, long-term outcome, mortality, functional outcome

## Abstract

**Background**: Ischemic stroke is the main cause of adult disability, with up to 25% of patients dying within the first year. In Germany, 4/10 patients receive in-hospital rehabilitation after acute stroke. Therefore, the aim of this study was to examine the association between modern neurological rehabilitation and the outcomes of patients with ischemic stroke. **Methods**: In our single-centre, prospective observational study, we included patients with ischemic stroke between March 2023–June 2025. Within the first year after discharge, we conducted telephone surveys, recording survival status, modified Rankin Scale score (mRS), and quality of life using EQ-5D-5L. Favourable outcomes were defined as mRS 0–2. Predictors of mortality and favourable outcomes after 12 months were calculated using multiple logistic regression. **Results**: We included 180 patients with ischemic stroke and complete one-year follow-up. Median age was 81 years, median Barthel Index at admission was 15 and median Charlson Comorbidity Index (CCI) was 4 points. Mortality during rehabilitation was 3.3% and 20.6% at 1 year. Only 10% achieved a favourable outcome after 12 months. Predictors of mortality were CCI (OR: 1.27 (1.01–1.61)) and discharge home (OR: 0.18 (0.06–0.48)). Predictors for favourable outcome were age (OR: 0.92 (0.87–0.97)), length of stay in rehabilitation (OR: 0.94 (0.91–0.97)) and weekly duration of neuropsychology (OR: 2.79 (1.27–6.66)). **Conclusions**: Multimorbid patients needing institutional care appear to have greater risk of death, while outcomes of younger patients, who needed less rehabilitation and had more consultation with neuropsychology, were associated with higher levels of independency after one year.

## 1. Introduction

In 2019, over 12 million people worldwide were diagnosed with a first-ever stroke [1], 80% of which were caused by ischemia. The majority (80%) of these patients suffer sensory and motor disability, especially hemiplegia [2]. Therefore, stroke is the leading cause of physical disability in adults [3]. In Germany, only 60% of patients show minor functional loss (mRS 0–2), while almost 20% need constant support (mRS 4–5) [4]. Furthermore, up to 21% of patients die within the first year after an ischemic stroke [5]. Meta-analyses report a decrease in mortality and an increase in quality of life after multidisciplinary stroke care, including rehabilitation [3,6]. Early rehabilitation starting 24 hours to two weeks after stroke has been demonstrated to improve functional ability and quality of life (QoL) [7,8,9]. However, in Germany, less than 40% of ischemic stroke patients received in-patient rehabilitation after discharge from hospital [2]. Only in Australia, New Zealand and the United Kingdom does rehabilitation for stroke patients seem to be consistently recommended in clinical guidelines, whereas in the United States, Canada and Europe it is not, leading to varying in-patient rehabilitation rates, from 13% in Sweden to 57% in Israel [10]. Therefore, the recovery of disabilities caused by stroke is a globally important task for rehabilitation professionals. In stroke literature, factors influencing patient outcomes after stroke are widely examined, especially for baseline data [11,12,13] and comorbidities [12,13,14,15,16,17,18] and even very specific treatment approaches [19,20,21] or biologic markers [22,23,24]. However, global outcome studies after neurologic rehabilitation are rare, often lacking information of rehabilitation data or only examining a specific therapy component, such as gait [25,26,27] or dysphagia [28,29]. Additionally, the majority of patient outcomes are measured in-hospital [30] or three months after stroke [31], though an important part of rehabilitation occurs up to six months after stroke [3,32]. Only recently have studies increasingly investigated long-term outcomes [33,34,35,36,37,38,39].

Therefore, this study examined long-term outcomes of patients one year after ischemic stroke and after modern neurological rehabilitation with a focus on mortality and global functional recovery. We hypothesized that the quality and intensity of specific rehabilitative therapies are associated with surviving and regaining high functional status one year after ischemic stroke.

## 2. Materials and Methods

### 2.1. Study Design and Subjects

This prospective, non-interventional study was conducted at a neurological in-patient rehabilitation centre in Germany that offers in-patient evidence-based and intensive neurological rehabilitation treatment to its patients. Importantly, no patients with a positive back-to-work prognosis insured via Deutsche Rentenversicherung are admitted to our rehabilitation centre. Between March 2023 and June 2025, consecutive patients over 18 years who provided informed consent to the study and had suffered a recent ischemic stroke were included. Patients who were under 18 years old, had not suffered a recent stroke, or had already been included in the study at an earlier time were excluded. Demographic and clinical data were collected at baseline and routinely over the period of rehabilitation. We recorded quality and quantity of all rehabilitation treatments applied to the patients. After discharge, we conducted telephone surveys at three, six, nine and twelve months and recorded survival status. To ensure as much consistency as possible and minimize inter-rater bias, the telephone calls were standardized [40] using a questionnaire and conducted by three interviewers who had received detailed instructions and rigorous training in the assessment tools used in this study. If patients withdrew consent to the study during rehabilitation or the follow-up period or could not be reached after three attempts, they were excluded from the study.

### 2.2. Assessments

During rehabilitation, the ‘Charlson Comorbidity Index’ (CCI) [41] at baseline was determined to assess multimorbidity. This score has been originally developed for oncologic and later validated for stroke patients [42,43]. Corresponding variables are myocardial infarction, heart failure, peripheral arterial disease, cerebrovascular disease, dementia, chronic obstructive pulmonary disease, connective tissue disease, ulcer, diabetes, renal and liver failure, hemiplegia, malignant tumours, and AIDS, each scored according to its severity.

To quantify autonomy in daily living, ‘early-rehab Barthel Index’ (ERBI) and ‘self-independence in neurologic-geriatric rehabilitation’ (SINGER) were assessed weekly. The ERBI is an extension of the Barthel Index (BI) [44,45], which originally describes dependency in activities-of-daily-living (ADL) with a maximum of 15 points achievable in 10 categories. If an ADL can be performed independently, 10 or 15 points are scored respectively, whereas complete dependency scores 0 points. The ERBI [46,47] additionally scores 25 or 50 negative points respectively for severe neurological deficits such as mechanical ventilation, tracheostomy, and dysphagia, in order to reduce floor effects [48]. The BI is validated for stroke patients [31,49,50]. SINGER was introduced as a tool to better track rehabilitation effects in neurological rehabilitation facilities. It demonstrated less floor and ceiling effects than BI [51], but nonetheless cannot display minor impairments [52]. It includes 20 items of ADL with six levels, ranging from ‘completely dependent needing professional help’ to ‘independent without assistive device’.

During follow-ups, the ‘European Quality of Life 5 Dimensions 5 Level Version’ Score (EQ-5D-5L) [53,54] was used to quantify quality of life. The score is composed of two parts. The first part describes health in five different dimensions (mobility, self-care, daily activities, pain/discomfort, and anxiety/depression), each with five possible options ranking how affected patients are (none, slightly, mildly, severe or extreme). The second part is a visual analogue scale (EQ-VAS) ranging from 0 (worse) to 100 (best). This score, like the previous EQ-5D-3L, exhibits ceiling effects [55]. There are multiple population reference data studies from various countries, e.g. Germany [56], including assessment of the elderly [57] and validation for stroke patients [58,59].

Functional independence was assessed employing ‘modified Rankin Scale’ (mRS), which was developed to evaluate dependence in daily living of stroke patients [60,61]. It ranges from 0 (no disability) to 6 (dead). The reliability of the mRS is moderate, as it suffers interobserver variability [62]. However, functionality can be predicted by mRS [63]. Following a common definition of a good functional outcome of ischemic stroke patients [64,65], dichotomized mRS score of 0–2 and 3–6 at 12 months were utilized to generate two reference groups for evaluation.

### 2.3. Statistical Methods

Categorical baseline characteristics are depicted as number and percentages and continuous variables as medians as well as first and third quantile (IQR). Spearman correlation analyses were conducted. Group comparison of survival and favourable outcome were assessed using simple logistic regression including Wald-chi-squared test for all variables. Based on β-coefficients of logistic regression, odds ratios (ORs) with 95% confidence intervals (CIs) were calculated. Simple logistic regression was performed with the following variables: age, sex, living alone, smoking, consumption of alcohol, CCI (included categories: myocardial infarction, heart failure, peripheral arterial disease, dementia, chronic pulmonal disease, ulcer, hemiplegia, tumour), cardiovascular risk factors (hypertension, atrial fibrillation, coronary heart disease, diabetes, dyslipidaemia, obesity, stroke history), TOAST criteria of stroke, NIHSS, level of hospital, revascularisation therapy, (duration of) tracheal cannulation, length of stay in acute hospital and rehabilitation, complications during rehabilitation, neurogenic oropharyngeal dysphagia at admission and discharge, ERBI at admission and discharge plus difference, SINGER at admission and discharge plus difference, therapy in rehabilitation (physiotherapy, occupational therapy, speech and deglutition training, music therapy, neuropsychology) in total hours and hours per week, device-supported gait training and discharge home. All variables with *p* ≤ 0.01 were entered into a multiple logistic regression analyses adjusted for age, sex, ERBI at admission and CCI. Due to high correlation (r > 0.6), ERBI at discharge as well as SINGER at admission and discharge were excluded from the model for survival and SINGER at admission and discharge for outcome analyses. All statistical calculations were performed using R (version 4.4.3, R Foundation for Statistical Computing).

## 3. Results

A total of 261 ischemic stroke patients were included in the study. Of these, eight were excluded either due to withdrawal of consent or lost-to-follow-up. A total of 180 patients reached 12-month follow-up outcome and were evaluated.

### 3.1. Baseline Characteristics

Median age was 81 years (IQR: 72–84) and 55% of patients were female. Prior to the event of stroke, around half of patients had lived at home by themselves, only 3% in a residential care facility. Most patients had at least two cardiovascular risk factors (Table 1). The most common risk factor was hypertension (81%), followed by dyslipidaemia (43%) and atrial fibrillation (37%) as well as diabetes mellitus (37%). The median CCI score was 4 (IQR: 3–5). By far the majority of strokes were caused by occlusions of the middle cerebral artery. According to the TOAST criteria, most strokes were caused equally by large-artery atherosclerosis and cardioembolism (37% each). Only 8% were caused by small-artery occlusions, and 18% were categorized as strokes of undetermined cause. More than half (56%) of acute stroke care was delivered in maximal care hospitals, with 40% of patients receiving revascularisation therapy. Median initial NIHSS score was 8 (IQR: 5–13.75) and the median length of stay in acute care was 11 days (IQR: 8–16). Due to severe dysphagia, 13 patients were supplied with a tracheal cannula.

### 3.2. In-Patient Neurological Rehabilitation

At admission, median ERBI was 15 (IQR: −15–35), median SINGER 20 (19–48.5) and median length of stay was 49 days (IQR: 33–66.25). During rehabilitation, we observed significant global functional improvement in our cohort: Median ERBI increased by 35 points and median SINGER by 29 points from admission to discharge (*p* < 0.001 each). Patients accumulated a considerable dose of multiple rehabilitative therapies (Table 2), most commonly physiotherapy and occupational therapy. The median total therapy time was 65.75 (43.2–95.4) hours; the median weekly therapy time was 9.4 (8.3–10.9) hours. Device-supported gait training with Lyra® and/or Lokomat® received 61 patients. Nine of 13 patients could be decannulated following severe dysphagia improvement. During rehabilitation, 9 (3.3%) patients died. Medical complications occurred in 69 patients (38.3%) with falls as the most common complication (n = 51, 28%). The second-most cause was pneumonia, which occurred in 15 patients, eight of them with aspiration pneumonia. One patient suffered a recurrent stroke. At the end of rehabilitation, 61% could be discharged home; the proportion of patients needing institutionalized help increased from 3 to 39%.

### 3.3. One-Year Follow-Up

Mortality increased from 8% to 21% between the first and last follow-up. Median mRS and EQ-5D-5L at 12 months were 4 (3–5) and 3 (2.4–3.6) respectively. Median quality of life was 50 (36.25–60). At 12 months, only 10% of patients showed a favourable outcome with mRS 0–2 and 70% poor outcomes with mRS 3–5. During the follow-up period, neither the mRS (*p* = 0.1) nor quality of life (*p* = 0.2) significantly changed. However, EQ-5D-5L showed a significant decrease from three to twelve months follow-up (*p* = 0.002).

**Figure 1:**
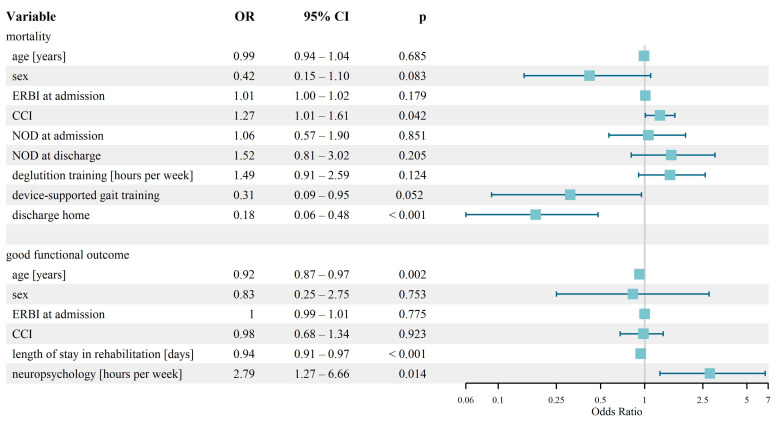
Results of multiple logistic regression model of mortality and good functional outcome. Abbreviations: OR, odds ratio; CI, confidence interval; ERBI, early-rehabilitation Barthel Index; CCI, Charlson Comorbidity Index; NOD, neurogene oropharyngeal dysphagia.

Single logistic regression analyses showed association between one-year mortality and high CCI score, heart failure, atrial fibrillation, higher degree of dysphagia measured by neurogenic oropharyngeal dysphagia (NOD) score at admission and discharge, lower ERBI score at discharge, lower SINGER score at admission and discharge as well as smaller improvement in SINGER, more hours of deglutition therapy per week, no device-supported gait training and discharge to a residential care facility. In multiple logistic regression model adjusted for age, sex, ERBI at admission and CCI only CCI and discharge home remained significant (Table 3, Figure 1). Higher CCI (OR: 1.27 (1.01–1.61)) was associated with higher mortality, while discharge home (OR: 0.18 (0.06–0.48)) was associated with lower mortality. There was a trend for decreased mortality risk if receiving device-supported gait training (OR: 0.31 (0.09–0.95)).

The following variables were dependently associated with a favourable outcome after rehabilitation: younger age, stroke not due to cardioembolism, a shorter length of stay in rehabilitation, higher SINGER score at admission and discharge, lower total therapy hours, fewer total hours of occupational therapy, higher hours of neuropsychology per week, application of device-supported gait training and discharge home. Multiple regression analyses for good functional outcome (Table 3 and Figure 1) showed positive association with hours of neuropsychology per week (OR: 2.79 (1.27–6.66)) and negative association with age (OR: 0.92 (0.87–0.97)) and length of stay in rehabilitation (OR: 0.94 (0.91–0.97)).

## 4. Discussion

This single-centre study of long-term outcomes after intensive stroke rehabilitation found a one-year mortality of 21% and a favourable outcome with high independency of mRS 0–2 in 10% of patients. Multiple logistic regression analyses revealed an association of mortality with higher level of comorbidity and discharge to an institutional care facility, whereas a good functional outcome was associated with younger age, a shorter length of stay in rehabilitation and more hours of neuropsychology per week.

The mortality rate of this study is equal to findings of another German study of Rücker et al., who evaluated case-fatality rates of first-ever ischemic stroke patients from 1996 to 2015 [5]. They found an overall probability to die one year after stroke of 21%. Rücker’s patients were younger (mean age 74 years) than our cohort and supposedly (admission NIHSS or ERBI not given) less severely affected than our in-patient-rehabilitation cohort. Acute stroke therapy has improved over the last years [66], leading to reduced post-stroke mortality and higher functionality [67] and might explain these equal rates despite different baseline characteristics. For instance, early post-stroke mortality (measured 30 days after ictus) has decreased during the last decade from 13.7% to 12.3% in Organisation for Economic Co-operation and Development (OECD) countries, ranging from 2.9% (Japan) to 20.5% (Latvia) in 2021 [68].

We used the Charlson Comorbidity Index (CCI) to figure expected one-year mortality in our cohort of patients. According to Charlson et al., a mean CCI of 4 in our stroke cohort would amount to a one-year mortality of 52% and even 85% when age is added as prognostic factor [41]. This predicted mortality rate is significantly higher than the 21% of our patients. A positive influence of therapies addressing hemiplegia, one category of the CCI which applies to almost every patient, could be a possible explanation. Furthermore, besides many factors including access to healthcare systems, availability of stroke units and early recanalization, one important factor might be the impact of high-intensity immediate post-stroke neurorehabilitation in general. Nam et al. could show that, in Korean patients, higher frequency (>40 session) of rehabilitation reduced long-term mortality in haemorrhagic but not in ischemic stroke patients [69]. Hsieh et al. demonstrated high-intensity rehabilitation to be significantly associated with reduced mortality compared to low-intensity rehabilitation [70]. In comparison to studies without given data on in-clinic rehabilitation, our post-stroke mortality was similar, though their patients were younger [35] and less affected [36]. Our mortality value was even lower than the 1-year-mortality value of 56% in moderate to severely affected ICU stroke patients [34] or than the mortality rate of 68% in ischemic and haemorrhagic strokes combined in China [71]. These results further emphasize a possible positive impact of rehabilitation on mortality. We hypothesize that in-patient neurological rehabilitation after ischemic stroke significantly reduces post-stroke mortality. Our study design, however, is not suitable to verify this hypothesis as there is no control group. In addition, we might underestimate true mortality of our stroke cohort since those patients dying early from their stroke would not be presented to our rehabilitation unit.

The association of comorbidity and mortality has been demonstrated by other authors as well. Fischer et al. concluded atrial fibrillation, coronary artery disease and diabetes significantly impacting stroke outcomes, but did not find and association with the CCI [72]. Several systematic reviews and meta-analyses reported higher age [11,12,13], cardiovascular risk factors [12,14,73] and smoking [74] to be associated with mortality, which this study could not emphasize. One possible explanation is the advanced age and similar distribution of cardiovascular risk factors in the cohort, and in the case of smoking, the small number of cases recorded.

Furthermore, this study found an association between discharge home status and reduced one-year mortality with a strong effect size (OR 0.18). Kimura et al. (n = 10,000) discovered similar results [75]. Other authors found associations between higher functional status and discharge home [76,77] as well as age and stroke severity as predictors of long-term institutional care [78].

There was a strong indication (*p* = 0.052) of lack of device-supported gait training to be associated with higher mortality, which would contradict findings of Mehrholz et al., who could not show any influence of electromechanical-assisted gait training on mortality [25].

The proportion of our patients with good 12-month functional outcome was low despite early and intensive rehabilitation. Kortelainen et al., for example, found almost twice our rate (17%) in more severely affected, but younger patients [34]. Higher age has been shown to be associated with unfavourable outcome using mRS < 3 [13,34,36] as well as BI < 15 [79]. This might explain our patients not achieving positive long-term outcomes after rehabilitation therapy. The same applies to baseline comorbidity [80,81], especially cardiovascular risk factors [13,14], and NIHSS at baseline [34,35,36], whose association with functional outcome this study could not emphasize, although other studies have demonstrated their influence.

Furthermore, pre-stroke dependency influences post-stroke outcomes as has been shown by Sennfält et al., who saw significantly more favourable outcomes in pre-stroke independent patients than in pre-stroke dependent ones [37]. Critically, we did not capture pre-stroke dependency, but due to advanced age and various comorbidities it might be reasonable to assume high pre-stroke dependency levels in our cohort. Additionally, there might be responders and non-responders to intensive neurorehabilitation as well as different recovery trajectory classes, which Grabowska-Fudala et al. evaluated to have different outcomes according to their baseline characteristics [82].

This study found association between favourable outcome and weekly consultation time with neuropsychology, whose positive effects on QoL have already been demonstrated in patients with attention deficit hyperactivity disorder and/or a mood disorder [83] and patients with various neurological deficits [84]. Thus, neuropsychological rehabilitation is recommended for patients with stroke or head injury [85]. This study did not find any further significant associations between other rehabilitation therapies and outcomes after stroke. In contrast, Hartley et al. and Huang et al. demonstrated physiotherapy positively influences physical activity and QoL [86,87]. Two Cochrane reviews found either unclear effectiveness of occupational therapy for cognitive impairment after stroke [88] or low evidence for improvement in ADL [89]. Another Cochrane review reported no significant influence on mortality or dependency after swallowing therapy, but a reduction in dysphagia [90]. Chiaramonte et al. found dysarthria to be significantly improved after speech rehabilitation [91].

In this study, simple logistic regression showed an association between mortality and higher amount of deglutition therapy per week as well as between favourable outcome and lower time of occupational therapy and therapy in total. These associations are not independent. It should be emphasized that patients suffering severe strokes get more intensive therapies to address their impairments. For example, simple regression further showed association between mortality and higher NOD. Patients suffering from dysphagia receive a higher amount of deglutition therapy to regain their loss of function and may have a higher risk of death because of their impairment including risk of aspiration pneumonia but not as a result of treatment. The same applies to favourable outcome and (occupational) therapy. While more frequent therapies might be associated with higher mortality within our study, this does not imply causality. Rather, based on our clinical experience, less received therapies often indicate a better functional status at admission, which is a predictor for a good functional outcome [92]. Conversely, severely affected patients have a greater risk of poorer functional recovery and lower QoL [93]. Therefore, these patients might require a greater number of therapies to address their needs, especially physio- and occupational therapy as restoration of motor skills is a central component of hemiplegia recovery.

Likewise, the association between shorter lengths of stay in rehabilitation and favourable outcome must be interpreted with caution. It is most likely due to severely affected patients requiring augmented therapy and considerable time in rehabilitation. Conversely, less affected patients had a higher chance of a favourable outcome even though they received less intensive rehabilitation. For example, Lu et al. found stroke severity as a predictor for prolonged stay in hospital and lack of outcome improvement in ischemic stroke patients staying more than 14 days in hospital [94]. Clark et al. showed increased rehabilitation time resulting in little to no difference in ADL and upper and lower limb activities, though motor impairment could be slightly improved. Additionally, there might be a threshold of time spend in rehabilitation, upon which outcomes will be improved [95]. The failure to reach that threshold might explain prolonged stays in rehabilitation care without significant improvement in functional levels. Currently, however, there is no recommended minimal daily amount of rehabilitation upon which functional improvement is reached.

Based on our results, we recommend that patients be discharged home rather than to a nursing home in our clinical practice. Furthermore, a broadened supply of device-supported gait training and neuropsychology should be taken into consideration as these therapies seem to improve patient outcomes the most.

### 4.1. Limitations

Notably, the high median age of 81 years in our patients indicates an elderly population typical for (Eastern) German patients in a rural area. Furthermore, only patients, who had suffered a sufficiently severe stroke requiring in-patient rehabilitation were included in this study. We can only speculate on the degree to which our results can be generalized to other populations worldwide. We assume geriatric populations who have access to a specialized acute stroke care system to be similar to our cohort. This includes stroke units and access to revascularisation therapies within short access routes. Outcomes in younger patients with more resilience might differ as well as in patients affected less or worse than ours, as our patients median NIHSS is 8 with only a small IQR of 8.75 (5–13.75). Additionally, comparability might be limited due to in-patient rehabilitation facilities in Germany, which are not standardly implemented in other countries [96].

Follow-up interviews were conducted by multiple trained interviewers, so inter-rater variability for EQ-5D-5L and mRS scores might be present. Data were collected from patients, their relatives or their legal representatives, influencing the subjective EQ-5D-5L and QoL.

Unlike most studies that include NIHSS as a covariate for predictor models, this study used ERBI at admission in logistic regression analyses. This is due to the limited number of patients (n = 136) NIHSS was assessed in acute care. Therefore, 25% of data sets would have been incomplete and unusable for logistic regression analysis. Secondly, the NIHSS is assessed on admission in acute care, so potential improvements during hospitalization are not accounted for. Since logistic regression indicates an association between an increased therapy time or length of stay and a higher risk of an unfavourable outcome, which is assumed to be linked to greater impairment, the ERBI score might not be the most valid method for evaluating patient impairment.

The assessment of functional status in our study differs between rehabilitation (ERBI and SINGER) and follow-up (EQ-5D-5L and mRS). All scores assess dependency in ADL, though slightly differently. ERBI and SINGER assess dependency in ADL rather detailed, while mRS only has four categories. EQ-5D-5L focuses on QoL. This leads to a limited comparability between dependency levels in rehabilitation and after one year.

Furthermore, we used a study design without a control group, as excluding patients requiring rehabilitation would be ethically unacceptable. Since the indication for rehabilitation is based on the severity of stroke, a control group with patients not receiving rehabilitation is likely to introduce new confounders. Subdividing the study cohort into patients receiving extensive and those receiving minimal rehabilitation would be possible in the future; however, it must be noted that more severely affected patients require more rehabilitation, thus again compromising the comparability of the cohorts. An expansion of this study to other rehabilitation centres might help improve data transferability.

### 4.2. Further Investigation

Further work needs to be done in order to identify responders and non-responders to intensive neurorehabilitation. Additionally, a separation in patients receiving different rehabilitation consultation time could improve stating direct causality between patient outcomes and rehabilitation therapies. As declining therapy to patients with clinical indication is not ethically acceptable, a division in groups receiving the minimally needed amount of rehabilitation and high-dose-rehabilitation-recipients might be possible. Adding pre-stroke dependency to assessed factors might improve the validity of the results.

## 5. Conclusions

This study found 10% of patients having a favourable outcome of high independence despite the severity of strokes, high baseline age and multimorbidity of the cohort. Younger age and extensive consultation with neuropsychology were associated with better outcomes after one year, while multimorbid patients and patients needing institutional care had association with increased mortality. The present predictors are consistent with current studies and the association of rehabilitation on patient outcomes was evaluated. However, a change in study design might be necessary to assess influence of rehabilitation with causality and improved transferability.

## Figures and Tables

**Table 1:** Baseline characteristics of ischemic stroke patients with 12-month follow-up (n = 180).

Variables	n_total_	n (%)	Median (Q_25_–Q_75_)
Demographic data			
age [y]	180		81 (72–84)
sex, female	180	99 (55.0)	
civil status	179		
solitarily		89 (49.7)	
partnership		90 (50.3)	
housing situation	179		
house, flat		173 (96.7)	
residential care facility		6 (3.3)	
Risk factors and medical history			
alcohol	114	25 (21.9)	
smoking	114	32 (28.1)	
hypertension	180	145 (80.6)	
atrial fibrillation	180	66 (36.7)	
coronary heart disease	180	24 (13.3)	
diabetes mellitus	180	66 (36.7)	
dyslipidaemia	180	77 (42.8)	
obesity	180	34 (18.9)	
stroke history	180	32 (17.8)	
CCI	180		4 (3–5)
Stroke and immediate care			
NIHSS at baseline	138		8 (5–13.75)
TOAST criteria	166		
LAA		61 (36.7)	
CE		61 (36.7)	
SAO		14 (8.4)	
SUD		30 (18.1)	
infarct localisation MCA	168	95 (56.5)	
length of stay in hospital [d]	178		11 (8–16)
revascularisation therapy	170	68 (40.0)	
tracheal cannula	180	13 (7.2)	
Rehabilitation			
length of stay [d]	180		49 (33–66.25)
ERBI admission	180		15 (−15–35)
ERBI discharge	171		50 (20–60)
ERBI improvement	171		30 (10–47.5)
SINGER admission	165		29 (19–48.5)
SINGER discharge	164		58 (42–72)
SINGER improvement	164		20 (9–31)
complications	180	69 (38.3)	
fall		51 (28.3)	
(aspiration-)pneumonia		15 (8.3)	
stroke recurrence		1 (0.6)	
deceased during rehabilitation	180	6 (3.3)	
discharge	175		
home		107 (61.1)	
short-term care		11 (6.3)	
residential care facility		47 (26.8)	
other		10 (5.7)	
1-year-follow-up			
mRS	180		4 (3–5)
1–2		18 (10.0)	
3–5		125 (69.4)	
6		37 (20.6)	
EQ-5D-5L	143		3 (2.4–3.6)
mobility			3 (2–3)
self-care			3 (2–3)
daily living			2 (1–3)
pain			3 (2–4)
anxiety			4 (3–5)
VAS-EQ	142		50 (36.25–60)

Note: n_total_ gives information about how often certain data were collected; n (%) gives the number and percentage of positive answers for numeric variables. Metric variables are presented as median with 25th and 75th quantile. mRS 0 was excluded because it did not apply to any patient. Points in EQ are defined as follows: 5—no restrictions, 4—mild restrictions, 3—moderate restrictions, 2—severe restrictions, 1—extreme restrictions/not able. VAS-EQ ranges from 0 to 100. Abbreviations: CCI, Charlson Comorbidity Index; NIHSS, National Institute of Health Stroke Scale; TOAST, Trial of Org 10172 in Acute Stroke Treatment; LAA, large-artery atherosclerosis; CE, cardioembolism; SAO, small-artery occlusion; SUD, stroke of undetermined cause; MCA, middle cerebral artery; ERBI, early-rehabilitation Barthel Index; mRS, modified Rankin Scale; EQ-5D-5L, European Quality of Life in 5 Dimensions 5 Levels; VAS-EQ, European Quality of Life visual analog scale.

**Table 2:** Summary of therapies received in rehabilitation.

Therapy	n	Total [h] Median (Q_25_–Q_75_)	Dose [h/w] Median (Q_25_–Q_75_)
physiotherapy	179	24.5 (13.8–38.5)	3.9 (3.2–4.6)
occupational therapy	180	17.5 (8.4–27.1)	2.7 (2.1–3.2)
speech training	153	4.0 (1.5–7.0)	0.6 (0.3–1.2)
deglutition training	136	3.8 (1.5–8.6)	0.6 (0.3–1.3)
neuropsychology	151	8.0 (4.5–14.3)	1.4 (0.8–1.8)
music therapy	98	4.5 (2.1–7.9)	0.6 (0.3–0.9)
total	180	65.75 (43.2–95.4)	9.4 (8.3–10.9)

Data presented as median and first and third quantile for total time in hours as well as dose per 7-day-week in hours per week. n gives the number of patients who received this therapy. Medians are based on patients, who received this therapy.

**Table 3:** Results of multiple logistic regression model of mortality and good functional outcome.

	OR	95% CI	*p*
one-year mortality			
age [years]	0.99	0.94–1.04	0.685
sex	0.42	0.15–1.10	0.083
ERBI at admission	1.01	1.00–1.02	0.179
CCI	1.27	1.01–1.61	**0.042**
NOD at admission	1.06	0.57–1.90	0.851
NOD at discharge	1.52	0.81–3.02	0.205
deglutition training [hours per week]	1.49	0.91–2.59	0.124
device-supported gait training	0.31	0.09–0.95	0.052
discharge home	0.18	0.06–0.48	**<0.001**
good functional outcome (mRS 0–2)			
age [years]	0.92	0.87–0.97	**0.002**
sex	0.83	0.25–2.75	0.753
ERBI at admission	1.00	0.99–1.01	0.775
CCI	0.98	0.68–1.34	0.923
length of stay in rehabilitation [days]	0.94	0.91–0.97	**<0.001**

Note: Adjusted for age, sex, ERBI and CCI. Indication of odds ratios (ORs), 95% confidence interval (CI) and *p*-value. All variables from single regression with *p* ≤ 0.01 were included. For mortality, SINGER score at admission and discharge as well as ERBI at discharge were excluded due to high correlation with ERBI at admission. For functional outcome, SINGER at admission and discharge were excluded due to high correlation with ERBI at admission. Abbreviations: OR, odds ratio; CI, confidence interval; ERBI, early-rehabilitation Barthel Index; CCI, Charlson Comorbidity Index; NOD, neurogene oropharyngeal dysphagia; mRS, modified Rankin Scale.

## Data Availability

Due to privacy and further investigations, we are not able to make our data publicly available.
